# Supplementary material for: CO2 Adsorption on the N- and P-Modified Mesoporous Silicas
Source: Nanomaterials (Basel). 2022 Apr 5;12(7):1224. doi: 10.3390/nano12071224 (PMC9000677; doi:10.3390/nano12071224)
Supplement: Supplementary file 1 [file nanomaterials-12-01224-s001.zip › nanomaterials-1639852-supplementary.pdf]

nanomaterials

Supplementary Materials

CO_2 Adsorption on the N- and P-Modified Mesoporous Silicas

Oyundari Tumurbaatar [1], Hristina Lazarova [1], Margarita Popova [1], Violeta Mitova [2], Pavletta Shestakova [1] and Neli Koseva [2,*]

1 Institute of Organic Chemistry with Centre of Phytochemistry, Bulgarian Academy of Sciences, Acad. G. Bonchev St., bl. 9, 1113 Sofia, Bulgaria; oyundari.tumurbaatar@orgchm.bas.bg (O.T.); hristina.lazarova@orgchm.bas.bg (H.L.); margarita.popova@orgchm.bas.bg (M.P.); pavletta.shestakova@orgchm.bas.bg (P.S.)
2 Institute of Polymers, Bulgarian Academy of Sciences, Acad. G. Bonchev St., bl. 103A, 1113 Sofia, Bulgaria; mitova@polymer.bas.bg
* Correspondence: koseva@polymer.bas.bg; Tel.: +35-988-729-7361

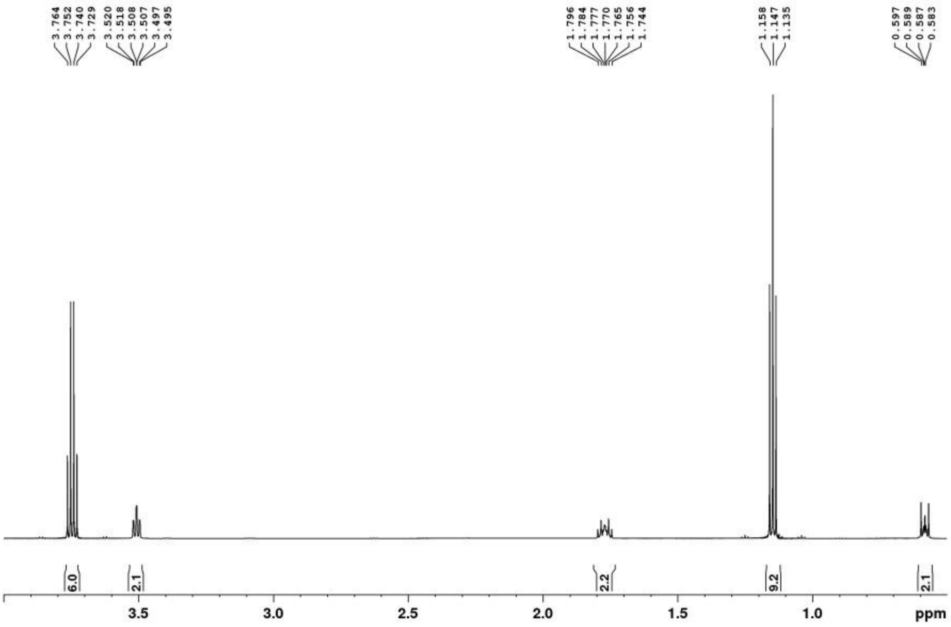

Figure S1. ^{1}H NMR spectrum of SAPTES in the spectral region from 4 ppm to 0.4 ppm.

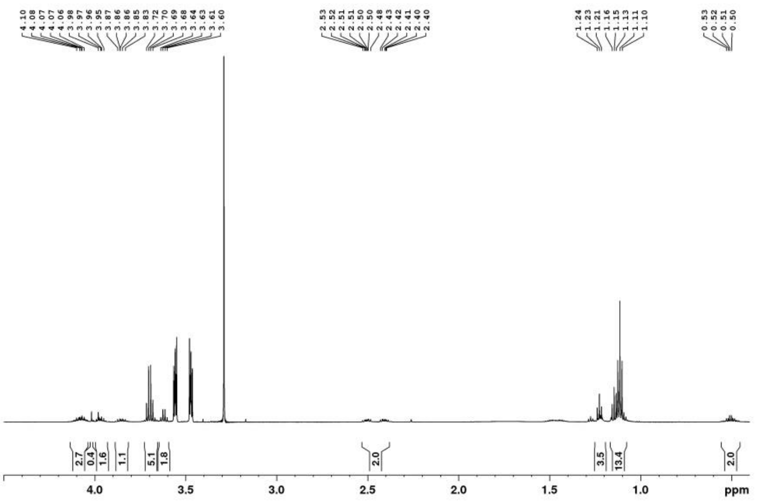

Figure S2. ^{1}H NMR spectrum of PAPTES in the spectral region from 4.5 ppm to 0.4 ppm.

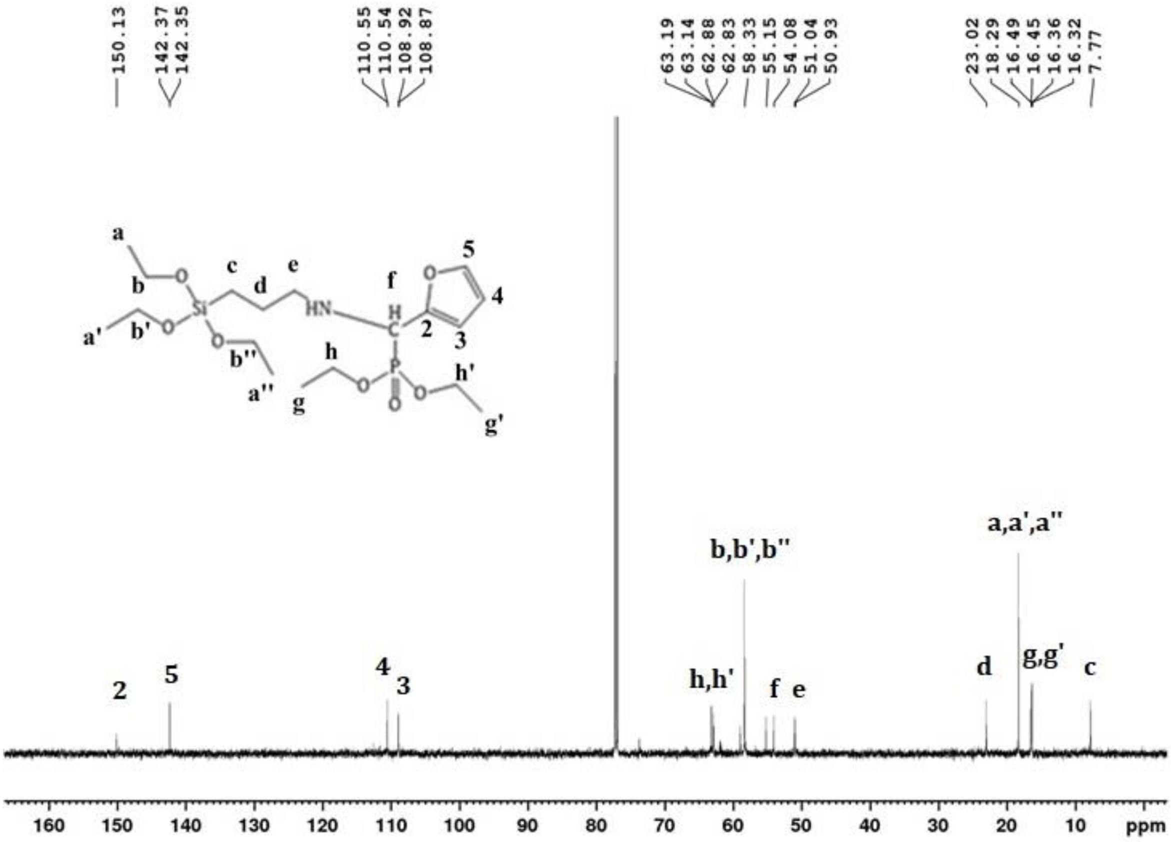

Figure S3. $^{13}C\{H\}$ NMR spectrum of PAPTES.

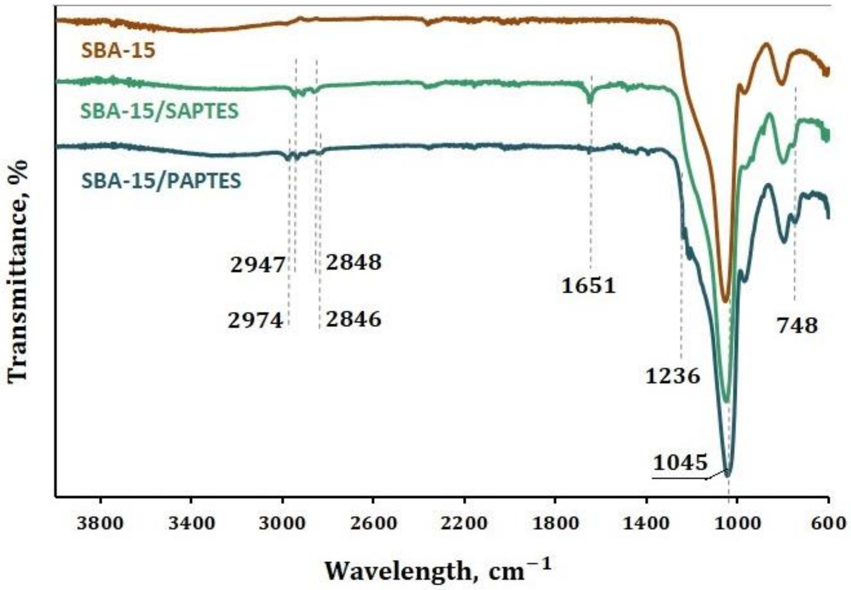

Figure S4. IR spectra of SBA-15, SBA-15/SAPTES and SBA-15/PAPTES.

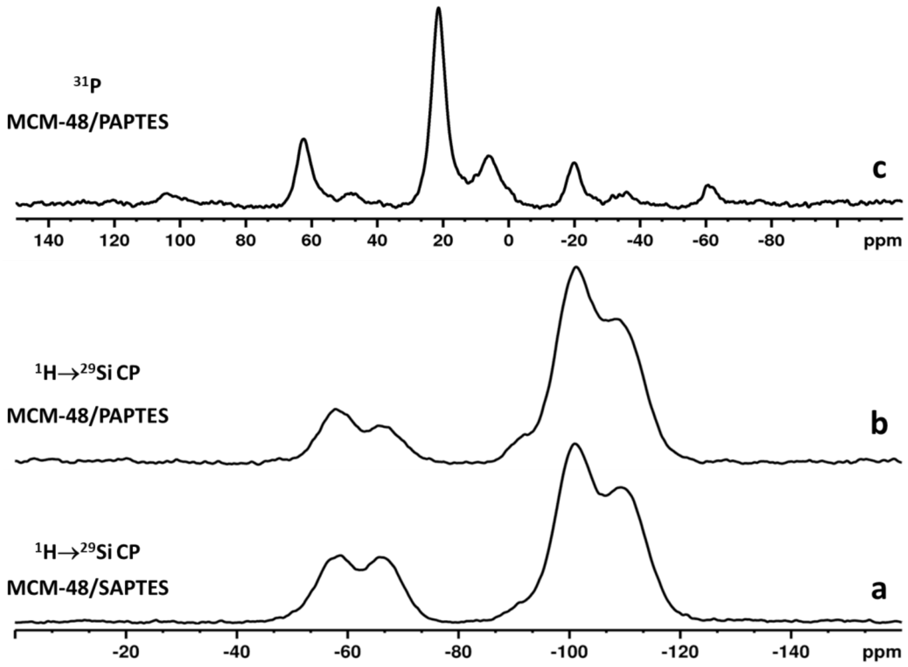

Figure S5. ^{1}H→^{29}Si CP MAS NMR spectra of (**a**) SAPTES and (**b**) PAPTES; (**c**) ^{31}P spectrum of PAPTES.

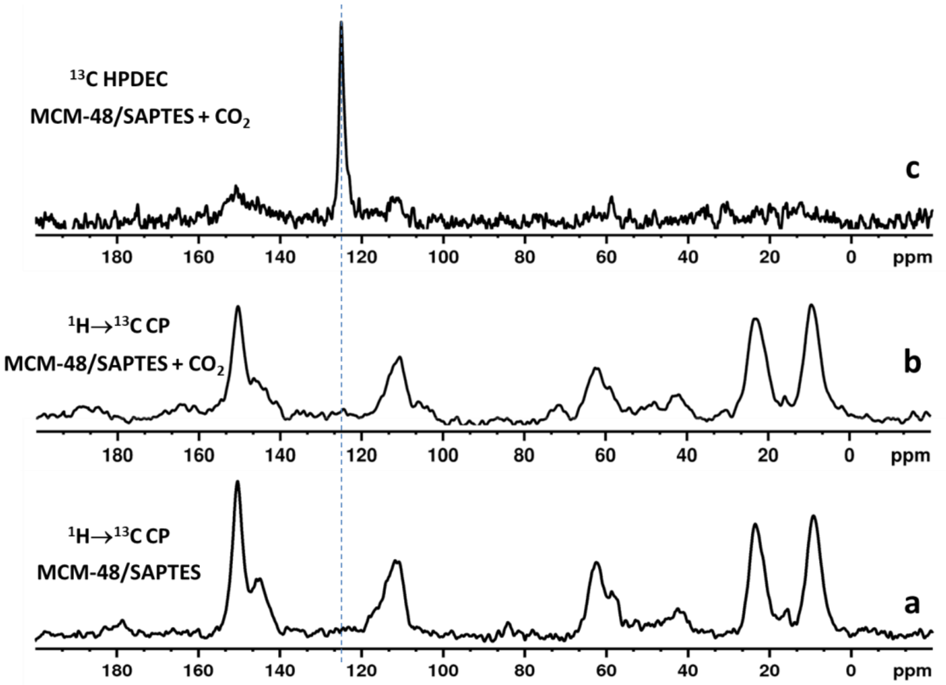

Figure S6. (**a**) ^{1}H→^{13}C CP MAS NMR spectrum of SAPTES; (**b**) ^{1}H→^{13}C CP MAS NMR spectrum of CO_2 loaded SAPTES; (**c**) ^{13}C HPDEC spectrum of CO_2 loaded SAPTES.

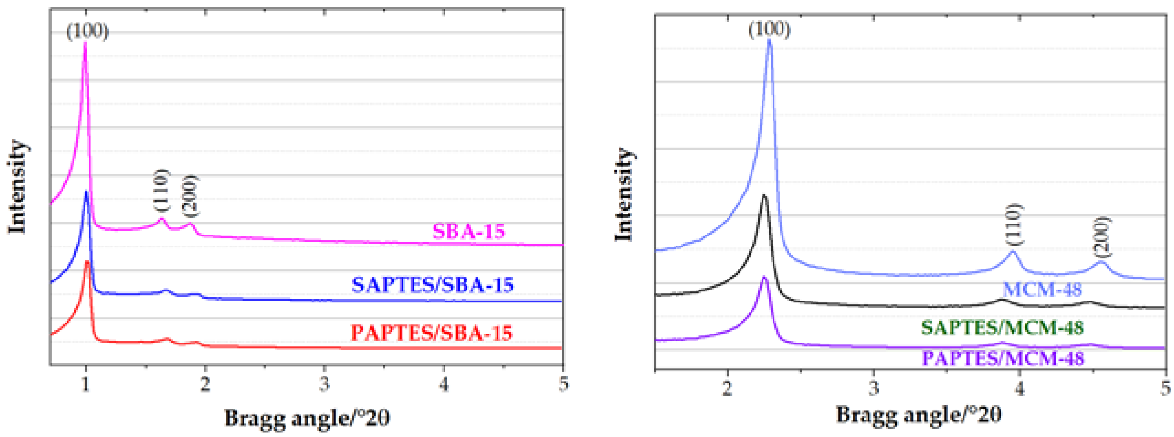

Figure S7. XRD of the initial and the modified SBA-15 and MCM-48 materials.

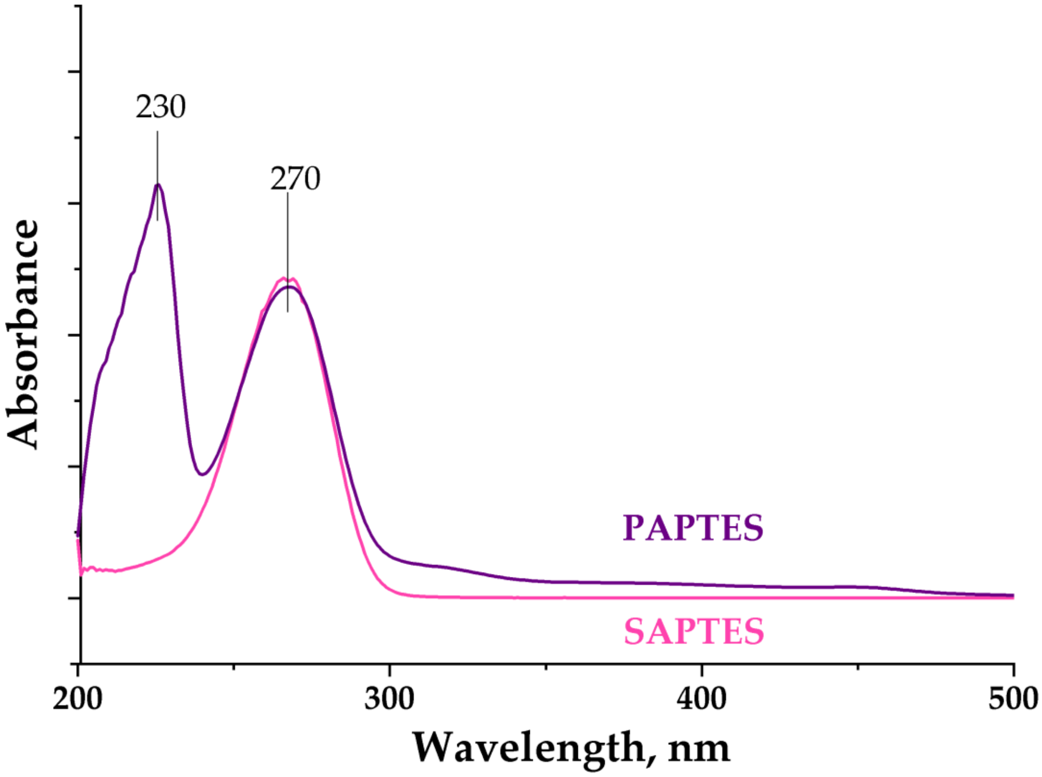

Figure S8. UV spectra of SAPTES and PAPTES.
